# Tungsten carbide–cobalt can function as a particle positive control for genotoxicity *in vitro* in specific cell lines

**DOI:** 10.1093/mutage/geaf021

**Published:** 2025-10-03

**Authors:** Michael J Burgum, Stephen J Evans, Ilaria Zanoni, Magda Blosi, Gareth J Jenkins, Shareen H Doak

**Affiliations:** In Vitro Toxicology Group, Faculty of Medicine, Health and Life Sciences, Institute of Life Sciences, Swansea University Medical School, Singleton Park, Swansea, Wales SA2 8PP, UK; In Vitro Toxicology Group, Faculty of Medicine, Health and Life Sciences, Institute of Life Sciences, Swansea University Medical School, Singleton Park, Swansea, Wales SA2 8PP, UK; CNR-ISSMC, Institute of Science, Technology and Sustainability for Ceramics, National Research Council of Italy, Via Granarolo, 64, 48018, Faenza, Italy; CNR-ISSMC, Institute of Science, Technology and Sustainability for Ceramics, National Research Council of Italy, Via Granarolo, 64, 48018, Faenza, Italy; In Vitro Toxicology Group, Faculty of Medicine, Health and Life Sciences, Institute of Life Sciences, Swansea University Medical School, Singleton Park, Swansea, Wales SA2 8PP, UK; In Vitro Toxicology Group, Faculty of Medicine, Health and Life Sciences, Institute of Life Sciences, Swansea University Medical School, Singleton Park, Swansea, Wales SA2 8PP, UK

**Keywords:** genotoxicity, in vitro, tungsten carbide–cobalt

## Abstract

Nanoparticle genotoxicity can be induced through several mechanisms, but there are currently no nanoparticle positive controls available for the evaluation of *in vitro* genotoxicity. Tungsten carbide–cobalt (WC/Co) has been proposed as one possible candidate. The aim of this study was therefore to investigate the genotoxic profile of WC/Co (Co 8% wt.) utilizing the cytokinesis-blocked micronucleus (CBMN) assay, the mammalian cell gene mutation test, and comet assay following a 24-hour exposure. This was conducted in human lymphoblast (TK6) and Chinese hamster lung fibroblast (V79–4) cells. No cytotoxicity was observed in the TK6 CBMN assay even when significant induction of micronuclei was observed at 100 μg/ml (2-fold over control). In contrast, V79–4 cells demonstrated no significant genotoxicity or cytotoxicity in the CBMN assay. In the gene mutation assay significant mutagenicity was observed in V79–4 cells at 100 μg/ml (2-fold over control). Cellular uptake of the WC/Co nanoparticles was not qualitatively detected in either cell type when investigated with transmission electron microscopy. No genotoxicity was observed in either cell type with the comet assay. The data generated indicates that WC/Co nanoparticles may be used as a positive particulate control in the CBMN assay when using TK6 cells only; whilst in the gene mutation assay it can be used as a positive control for V79–4 cells. However, its use as a particle positive control is only possible when applying the highest test concentration of 100 μg/ml.

## Introduction

Standard *in vitro* genotoxicity assays with associated Organization for Economic Co-Operation and Development (OECD) test guidelines (TGs), such as the micronucleus assay, chromosome aberration assay, and mammalian cell gene mutation tests were originally designed and for chemicals. However, these tests are subject to limitations when evaluating the genotoxicity of nanoparticles and nanomaterials, which compromises their robustness [1–4]. Within the current TG’s there is a lack of clarity pertaining to the handling of nanomaterials (preparation, sonication, dispersion, dose range), but of greater concern are issues surrounding the most appropriate exposure time and assay interference. For example, with the cytokinesis-blocked micronucleus (CBMN) assay, the concentration and exposure schedule and time of cytochalasin B need modification when evaluating nanomaterials. There has therefore been increasing emphasis on the need to adapt the OECD TGs to account for nano-specific considerations [5, 6]. In 2022 an OECD Guidance Document (GD No. 359) was published which provides recommendations and best practices for conducting the *in vitro* micronucleus assay with nanomaterials [7]. The aforementioned GD No. 359 provided data to indicate that tungsten carbide–cobalt (WC/Co) may act as a positive particle control in genotoxicity testing. This addresses a major concern around the current lack of a convincing nanoparticle positive control for genotoxicity testing [2, 3, 8–10].

Whilst WC/Co nanoparticles are already recognized as a workplace inhalation hazard by the National Institute of Occupational Safety and Health, the fundamental relationship between exposure, toxicity and subsequent progression towards disease is unclear [11]. The use of WC/Co as a positive particle control has been trialled in the scientific literature with notable examples published in 2014 and 2015 which highlight that these particles could serve as a positive control in select cell lines only [12, 13]. These studies highlight that WC/Co particles can induce genotoxicity in human lymphoblasts and L5178Y cells in both the micronucleus and the mouse lymphoma assay (MLA-TK) at concentration ranges up to 100 μg/ml. Moche *et al* report that in human lymphocytes and L5178Y cells point mutations, micronuclei induction and oxidative stress were observed. Whilst setting an excellent precedent, further work is required in other OECD-validated cell types to populate the data gaps which currently exist. For WC/Co nanoparticles, it is unclear what forms of DNA damage can be induced i.e. point mutations, double strand and/or single stranded DNA breaks. The aim of this study was therefore to determine if WC/Co could act as a reliable particle positive control in the *in vitro* alkaline comet assay, the *in vitro* mammalian cell gene mutation test (at the HPRT locus) and the *in vitro* CBMN assay.

## Materials & methods

### Preparation of the test materials

The tungsten-carbide-cobalt, (8% wt. WC/Co, <200 nm, 99.5% LOT#5561–072018, Nanostructured & Amorphous Materials Inc., USA), was weighed, suspended and sonicated according to the NANoREG protocol [14]. The nanoparticles were weighed using the OHAUS Explorer Semi-Micro Balance housed in a WAYSAFE (#GP1540). The positive chemical control, methyl methanesulfonate (MMS, #129995-5G) was purchased from Sigma-Aldrich (Sigma, UK) and diluted to working concentrations in water and complete cell culture medium.

### Acellular static dissolution of WC/Co nanoparticles

Static dissolution measurements were performed to assess the release of tungsten from nanoparticles at established incubation time points. The dispersion was diluted into the relevant medium (at concentrations of 20 and 100 μg/ml), and incubated in a thermal bath for 1 and 24 hours at 37°C. The ion release was calculated as the ratio between the dissolved ions after ultrafiltration and the total tungsten (tungsten-based NPs and free tungsten) present in the initial suspension, expressed as a percentage. Results from inductively coupled plasma optical emission spectrometry (ICP-OES) were reported as the average of three independent measurements with relative standard deviation (RSD). The ionic release was assessed by inductively coupled plasma (ICP) optical emission spectrometry using an ICP-OES 5100—vertical dual view apparatus (Agilent Technologies, Santa Clara, CA, USA) coupled with OneNeb nebulizer and equipped with an Autosampler. Argon was selected as the internal standard (wavelength 420 nm). The measurements were calibrated using calibration curves with a correlation coefficient limit > 0.999. The calibration fit was linear, including a blank in calibration. The precision of the measurements for the analysis expressed as the ratio of the standard deviation to the mean (RSD) in percentage, was always <5%. The limit of detection at the operative wavelength was 0.01 μg/ml for tungsten. Before each analysis, samples were acid digested, adding 10% v/v of nitric acid. Calibration curves were obtained in the range of 0.01–100.00 μg/ml, using standards prepared in medium and applying the same digestive procedure of samples.

### Nanoparticle characterization by dynamic light scattering

The hydrodynamic diameter of the WC/Co nanoparticles at each test concentration in both cell medium types was characterized by dynamic light scattering (DLS) using the Malvern Zetasizer Pro-Blue and software package ZS Explorer using Malvern ZEN 0040 low volume cuvettes. A total of three replicates were recorded each constituted of 5 runs each. The polydispersity index (PI) was also reported on provide an indicator of the width of the distribution within the data. The zeta potential of the WC/Co was also characterized in both cell culture medium types using the disposable Malvern Folded Capillary cell (#DTS1070) set to run at 37°C with three replicates per condition.

### Nanoparticle characterization by electron microscopy

Characterization of the WC/Co nanoparticles by transmission electron microscopy (TEM) consisted of first preparing the nanoparticle stock as detailed above to 2.56 mg/ml and then diluting to 100 μg/ml and drop casting 100 μl onto a carbon film, copper TEM grid (Agar Scientific). For analysis using the scanning electron microscope (SEM) the raw-state WC/Co nanoparticles were mounted onto adhesive carbon tape on an aluminium stub and loaded into the SEM. The SEM was used to gather large agglomerate Feret diameter data and the TEM used to measure the individual particle Feret diameter. The analysis tool used was the freely available ImageJ with all subsequent Feret diameter data transferred to GraphPad Prism to generate a histogram for both microscopic techniques. The SEM analysis was performed using the Hitachi Ultra High-Resolution field emission (FE)-SEM (S-4800 running at 10 kV). The TEM analysis was performed using the FEI Talos F200X G2 TEM operated at 200 kV with an FEI Super-X energy dispersive X-ray (EDS) detector system and a Ceta 16 M camera.

### Cell culture; human lymphoblastoid (TK6) cells

The TK6 cells (LOT #17E062) were purchased from the European Collection of Authenticated Cell Cultures. The cells were cultured in Roswell Park Memorial Institute (RPMI) 1640 (Gibco, UK) supplemented with 10% Horse serum (HS) and 1% L-glutamine. The TK6 cells were sub-cultured for one to two weeks prior to toxicology testing, checking regularly for changes to morphology. The TK6 cells were maintained at a concentration of between 2x10^5^ and 1x10^6^ cells/ml in T75 flasks.

### Cell culture; Chinese hamster lung fibroblast (V79–4) cells

The V79–4 cells LOT #70011007 purchased from American Type Culture Collection (ATCC) and were cultured in Dulbecco's Modified Eagle Medium (DMEM) 5546 containing 2 mM L-glutamine and supplemented with foetal bovine serum to a final concentration of 10% and 100 μg/ml penicillin and 100 μg/ml streptomycin (1% final concentration, respectively). The V79–4 cells were cultured to 80% confluency before being routinely sub-cultured in T75 flasks. When V79–4 cells required passaging, 4 ml of trypsin–ethyl-enediaminetetraacetic acid (EDTA) was added per flask to detach cells, an equal volume of complete culture medium was added to deactivate the trypsin, and centrifugation was always performed at 230 g for 5 minutes unless stated otherwise.

### *In vitro* cytokinesis-blocked micronucleus assay

The *in vitro* CBMN assay was followed and performed as described by the OECD GD No. 359 [7]. Briefly, the cells were seeded at 1.0 x 10^5^ cells/ml in T25 flasks (10 ml) and left to incubate overnight at 37°C. The following day the cells were counted then treated with WC/Co and MMS (TK6; 0.014 mM, V79–4; 0.1 mM) for 24-hours, complete cell culture medium was added to serve as the negative (vehicle) control. Following the exposure period, the cells were counted, before being centrifuged and washed with phosphate-buffered saline (PBS). The cells were then re-seeded into fresh T25 flasks in cell-culture medium containing 6 μg/ml of cytochalasin B for 24-hours. On the day of harvesting, the cells were then washed twice with pre-warmed PBS and centrifuged at 230 g; the cells were then resuspended in between 10 ml of PBS to be cyto-centrifuged (200 g for 5 minutes) onto glass microscope slides using a Shandon Cytospin. The cells were fixed by submerging the glass slides into ice-cold 90% methanol for 20 minutes at −20°C. The slides were then stained with filtered 20% Giemsa solution (pH 6.8 Gurr buffer) for 8 minutes. The slides were then laid flat before a single drop of DPX-mount was pipetted onto the cytodot and covered with a 22 x 22 mm coverslip and left to dry in a fume hood overnight. Three slides were prepared per concentration, per biological replicate whereby 1000 binucleated cells were scored per slide. Three biological replicates were performed in total.

### *In vitro* mammalian cell gene mutation assay—V79–4 cells

The *in vitro* mammalian cell gene mutation assay was conducted as described by the OECD TG No. 476 for both cell lines [15]. The V79–4 cells were first trypsinized using Trypsin–EDTA before 1x10^6^ cells were seeded per 80 cm^2^ petri dishes for each concentration and control groups. The V79–4 cells were then exposed to WC/Co and MMS at 0.1 mM for 24 hours. Following the exposure period, the cell medium was aspirated, and the cells washed twice with PBS. To check the viability a stock solution of 1x10^5^ cells/ml was prepared. An aliquot of this stock was diluted down to 1x10^3^ cells/ml before 200 μl was added to an 80 cm^2^ plate. The cells were then cultured between 5 and 7 days allowing small colonies (50 cells in diameter) to form. These were then stained with 1% methylene blue for 20 minutes before being rinsed with water and air-dried. The remaining stock of cells can be re-seeded into T25 flasks (1x10^6^ cells per plate/flask) from each exposure condition. These cells were sub-cultured every 2–3 days. Seeding cells for the assessment of mutation frequency and concurrent plating efficiency (cytotoxicity) was performed as follows. All sub-cultured dishes were trypsinized and a suspension of 1x10^3^ cells/ml was prepared per condition. For the plating efficiency, 100 μl of the 1x10^3^ cells/ml stock was seeded into x6 (replicates), 80 cm^2^ petri dishes and cultivated for 5–6 days before the colonies were stained with 1% methylene blue. For mutation frequency, 2x10^5^ cells were seeded (5 ml of stock) into respective 80 cm^2^ petri dishes, each plate was topped up to 10–15 ml with complete culture medium. After 3 hours of incubation and cellular adherence to plates, 0.1 ml of 500 mg/ml 6-TG was added to each plate. These plates were incubated for 10 days before being stained with 1% methylene blue and scored. The remaining cell stock was re-seeded to perform the fourth sub-culture of the assay which equates to the second mutation frequency plating procedure. Here, the cells were cultured in 80 cm^2^ petri dishes (three dishes per condition) at 2x10^5^ cells per dish. These were routinely maintained for a further 2 days before repeating the mutant selection and concurrent plating efficiency procedure detailed above.

### *In vitro* mammalian cell gene mutation assay—TK6 cells

The TK6 cells were purified with hypoxanthine, aminopterin, and thymidine (Sigma #H0262) medium at 5x10^5^ cells/ml in 50 ml for 3 days (final concentration of 2x10^−4^ M hypoxanthine, 8x10^−7^ M aminopterin and 3.5x10^−5^ M thymidine), replenishing the medium on day two. The TK6 cells were centrifuged at 230 g for 5 minutes and washed in 10 ml of PBS, centrifuged once more before being resuspended at 4 x 10^5^ cells/ml in 50 ml of HT medium (hypoxanthine and thymidine to a final concentration of 2x10^−4^ M hypoxanthine and 3.5x10^−5^ M thymidine) (Sigma H0137) medium for 24 hours. Following centrifugation at 230 g for five minutes, the TK6 cells were resuspended in 200 ml complete cell culture medium for three days, replenished at Day 2. Following HPRT mutant cleansing, the TK6 cells were centrifuged at 230 g for 5 minutes and resuspended in fresh culture medium at 5x10^5^ cells/ml (10 ml volume) in T25 flasks for 24-hour exposures to WC/Co and MMS (TK6; 0.014 mM). Negative control consisted of the addition of an equal volume of complete cell culture medium to the cells. Following the exposure, TK6 cells were centrifuged at 230 g for five minutes and re-suspended in 10 ml PBS, this wash step was repeated once more. TK6 cells were then suspended in 10 ml of fresh culture medium and incubated at 37°C with 5% CO_2_. TK6 cells were then maintained at a concentration of 1.25 x 10^5^ cells/ml (10 ml volume), being routinely passaged on Days 1, 3, 5, and 7-post exposure by centrifugation at 230 g for five minutes. On Day 9 post-exposure the volume was increased to 20 ml of complete cell-culture medium and on Day 11, this was increased to 50 ml of complete cell-culture medium. At 13 days post-exposure, TK6 cell stocks were prepared for each sample group at 4x10^5^ cells/ml (100 ml volume) for mutation frequency plating, and 100 cells/ml for plating efficiency, sufficient to plate x10 MF and x10 PE for each sample. Thus, 4x10^4^ cells/well and 10 cells/well were seeded in 100 μl for MF plates and PE plates respectively. Immediately prior to plating of mutation frequency, 6-TG (Sigma, A4882) was added to each stock flask for a final concentration of 0.6 μg/ml. The 96-well plates were then incubated undisturbed at 37°C with 5% CO_2_ for 14 days. After 14 days of incubation, plates were scored for colony formation whereby a single colony is >20 cells in diameter. The outer wells of each 96-well plate were not scored (60 wells per plate were scored). The cytotoxicity and mutation frequency data were generated using equations (1)–(3) (below); whereby the selective conditions were labelled as: 𝑋_S_=Number of wells without colonies, 𝑁_S_ = Total number of wells (non-selective conditions). 𝑋_0_ = Number of wells without colonies and 𝑁_0_ = Total number of wells. A total of three biological replicates were conducted.


(1) \begin{equation*} \mathrm{Plating}\kern0.17em \mathrm{Efficiency}\%\left(\mathrm{PE}\right)=-\mathrm{Ln}\;\left({X}_o/{N}_o\right)\times 100 \end{equation*}

(2) \begin{equation*} \mathrm{Cell}\ \mathrm{viability}\ \left(\%\right)=\frac{PE}{PE\ of\ control}\times 100 \end{equation*}

(3) \begin{equation*} \mathrm{Mutant}\ \mathrm{frequency}\ \left(\mathrm{MF}\right): MF=\frac{- Ln\ \left(\frac{X_s}{N_s}\right)}{- Ln\ \left(\frac{X_o}{N_0}\right)}\ \mathrm{x}\ \mathrm{DF} \end{equation*}

\begin{align*} & \mathrm{Dilution}\ \mathrm{factor}\ \left(\mathrm{DF}\right) \\& \quad = \frac{\left(\mathrm{No}.\ \mathrm{of}\ \mathrm{initial}\ \mathrm{cells}\ \mathrm{per}\ \mathrm{well}\right)\ \mathrm{non}-\mathrm{selective}\ \mathrm{conditions}\ }{\left(\mathrm{No}.\ \mathrm{of}\ \mathrm{initial}\ \mathrm{cells}\ \mathrm{per}\ \mathrm{well}\right)\ \mathrm{selective}\ \mathrm{contitions}\ } \end{align*}

### *In vitro* alkaline comet assay

The comet assay methodology applied in this study follows the standard operating procedure described in the 2022 publication by El Yamani, which utilized a high throughput approach and considers nanoparticle interference testing [16]. The TK6 cells were seeded into Greiner 96-well plates at 15x10^3^ cells/well in 100 μl and exposed to WC/Co and the positive molecular control of MMS (TK6; 0.014 mM, V79–4; 0.1 mM) the same day. To achieve the correct concentration of test agent, the WC/Co were made up at twice concentrated in 100 μl and added straight to the cells in the plate (final volume in each well was 200 μl). The V79–4 cells instead were seeded at 15 x 10^3^ cells/well in 200 μl and left to adhere overnight. The following day, the V79–4 cells were exposed to the WC/Co and MMS (this time the final concentration made up in 200 μl and added directly to the cells) for 24-hours. Following the exposure period, the cells were washed and harvested (as explained in the cell culture section) and 50 μl of cell suspension from each well transferred to a clean well of the 96 well plate. To evaluate the cytotoxicity in parallel to the *in vitro* alkaline comet assay, a Trypan blue exclusion assay was performed for each biological replicate at every concentration. Following the 24-hour exposure to WC/Co and MMS (TK6; 0.014 mM, V79–4; 0.1 mM) 10 μl of cell suspension was removed from each well and mixed 1:1 with an equal volume of sterile filtered (0.4%) Trypan blue (ThermoFisher, UK #15250061). A 10 μl aliquot of this suspension was then loaded into a Haemocytometer and a viable: non-viable cell count was performed on four corners of the grid to produce a ratio of live: dead cells; live cells/(live + dead cells). This was then converted to cell viability (%) by multiplying by 100. To prepare the cell-gel dots on pre-coated normal-melting point (55°C) agarose (0.5% in distilled water) slides, 150 μl of pre-melted low-melting point (37°C) agarose (0.8% in PBS) was mixed with 50 μl of cell suspension before 10 μl of this was pipetted onto the chilled, pre-coated microscope slides. A total of eight gel dots were prepared per slide after which the slides were incubated at 4°C to stabilize the gels. All slides were transferred to cold lysis solution (2.5 M NaCl, 0.1 M EDTA, 10 mM Tris, and 10% Triton X-100 adjusted to pH 10 using 8 M NaOH) and incubated overnight at 4°C. Following the lysis period, the slides were immersed in alkaline solution (0.3 M NaOH, 1 mM EDTA) for 20 minutes followed by electrophoresis at 1 V/cm for 20 minutes (300 mA). The slides were then washed in PBS followed by distilled water and then fixed in a series of ethanol washes at 70%, 90%, and 100%. When ready to score, the gel dots were stained with SYBR™ Gold nucleic acid gel stain (ThermoFisher, UK) diluted in TE buffer (10 mM Tris–HCl, 1 mM Na_2_EDTA, and adjusted to pH 7.5–7.8). The slides were covered with a coverslip and examined via fluorescence microscopy using the Comet IV software (Perceptive Instruments) where the %tail DNA in 150 comets were analysed per biological replicate (50 cells per gel), (*n* = 3). The formamidopyrimidine-DNA glycosylase (FPG)-modified comet assay was performed with the inclusion of a post-lysis incubation with the FPG enzyme (NorgenoTech, Norway). The FPG enzyme acts by converting oxidized purines within the DNA into strand breaks which would be observable as an increase in %DNA in the comet tails. Following lysis, the FPG-labelled slides were incubated with FPG enzyme diluted in a buffer of 40 mM HEPES, 0.1 M KCl, 0.5 mM EDTA, 0.2 mg/ml bovine serum albumin (BSA) adjusted to pH 8.0 and incubated at 37°C for 30 minutes. Following enzymatic treatment, these slides underwent the same procedure as the non-FPG-treated slides detailed above (incubation in alkaline solution and subsequent electrophoresis). The data pertaining to FPG-treated cells was then presented as total strand breaks (SB + FPG). A total of 150 comets per biological replicate were analysed using the Comet IV software (Perceptive Instruments), (*n* = 3).

### Evaluation of uptake by transmission electron microscopy

The TK6 cells were exposed to WC/Co at a concentration of 20 μg/ml for 24-hours. Subsequently, 1 x 10^6^ cells were fixed, embedded, and sectioned via ultramicrotomy as detailed by Wills *et al* [17]. The image analysis was performed using the Thermo Fisher Scientific FEI Talos F200X scanning/transmission electron microscope (S/TEM) operating at an accelerating voltage of 200 kV. which combines high-resolution STEM and TEM imaging with energy dispersive X-ray spectroscopy (EDX/EDS) signal detection. An Oxford Instruments INCA 350 EDX system/80 mm X-Max SDD detector was used to measure the EDX spectra, and the images were captured on a Gatan Orius SC600A CCD camera.

### Statistical analysis

All data is presented as the mean ± the standard deviation (SD). Statistical analysis was performed in GraphPad Prism software version 8.4.3 (GraphPad, USA). Data were checked for normality using the Shapiro–Wilk test. For normally distributed data, a one-way analysis of variance was performed with post hoc Dunnett’s multiple comparisons applied to evaluate pairwise statistical significance between the control group and the test concentrations. If data was not normally distributed, a Kruskal-Wallis test was used. The positive control data of MMS was compared to the negative control using a standard t-test, where ^*^P ≤ 0.05, ^**^P ≤ 0.01, ^***^P ≤ 0.001, ^****^P ≤ 0.0001.

## Results

The genotoxic profile of WC/Co nanoparticles was assessed in TK6 and V79–4 cells to determine its potential use as a particle control in future *in vitro* genotoxicity studies. Prior to testing, the *in-situ* physico-chemical features were characterized using DLS, Zeta potential analysis, dissolution assessment, and SEMand TEM.

**Figure 1:**
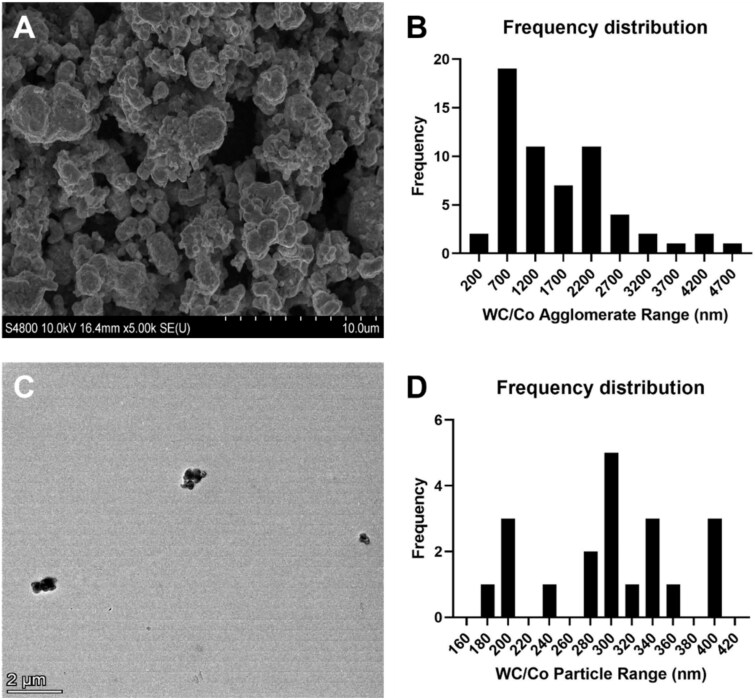
Electron micrographs and corresponding size distribution histogram of the Feret diameter for WC/Co particles. The particles (*n* = 60) were imaged using the SEM (A) and the subsequent particle size measured and plotted as a size distribution histogram in panel B. The TEM micrograph (panel C) and subsequent particle (*n* = 20) size distribution histogram is presented in panel D. Given there were fewer particles present for the TEM analysis a smaller ‘*n*’ number was collected for analysis.

To provide an indication of the WC/Co particle size in the stock solution (following sonication) and at a representative exposure concentration (20 μg/ml) both SEM/TEM were utilized. The resolution from both techniques was required due to the greater agglomeration and sheer particle number at 2.56 mg/ml when compared to the exposure concentration of 20 μg/ml. To provide a robust analysis of particle diameter, the software ImageJ was used to individually measure the Feret diameter. The particle size was investigated with the TEM (which could identify individual WC/Co particles) whilst the larger particles and particle agglomerates were measured using the SEM (Fig. 1). Analysis using the SEM highlighted a mean Feret diameter of 1594.9 ± 1011.449 nm whilst using the TEM to measure the smaller particulates the mean Feret diameter was calculated to be 299.5 ± 68.8 nm, which was close to the manufacturer’s stated size. In processing the data, the minimum Feret diameter on the TEM was 174 nm and the upper limit was 404 nm. Elemental analysis by EDS was also performed to determine the presence of impurities, none of which were detected (Supplementary Information, Fig. S2).

The hydrodynamic diameter of the WC/Co was characterized at each test concentration in both complete cell culture medium types for TK6 and the V79–4 cells. This was performed to correlate dose–response to particle and agglomerate size in complete cell culture medium. This data is summarized in Table 1. A concentration-dependent increase in the hydrodynamic diameter of the WC/Co nanoparticles was observed in both cell culture medium types. The data demonstrated that as the concentration of WC/Co is increased the agglomerate sizes increase dramatically up to 100 μg/ml, where the agglomerates are consistently over 1 μm in hydrodynamic diameter. The zeta potential of the nanoparticles in both cell culture medium types were observed to be consistently in the range of −4.1 and − 13.6 mV. The PI suggests that the WC/Co nanoparticles were unstable in suspension and formed multimodal families of agglomerates.

**Table 1:** Summary of the nanoparticle hydrodynamic diameter (Z-average), zeta potential and polydispersity index (PI) in complete RPMI 1640 and DMEM 5546 medium.

Concentration of WC/Co (μg/ml)	Z-average (nm) RPMI 1640	Zeta potential (mV) RPMI 1640	PI RPMI 1640	Z-average (nm) DMEM 5546	Zeta potential (mV) DMEM 5546	PI DMEM 5546
0	89.15 ± 90	−3.1 ± 0.7	0.64 ± 0.3	35.99 ± 1.6	−11.9 ± 2.4	0.8 ± 0.01
10	352.7 ± 251.4	−4.1 ± 1.3	0.3 ± 0.07	145.5 +/− 99.9	−8.25 ± 0.6	0.43 ±0.4
20	511.3 ± 226.2	−13.6 ± 2.4	0.38 ± 0.13	571.6 ± 220.2	−9.74 ± 1.3	0.41 ± 0.1
50	686.4 ± 152.9	−8.8 ± 0.7	0.46 ± 0.09	744.6 ± 276.5	−10.2 ± 1.2	0.52 ± 0.15
100	1253.7 ± 434.4	−10.3 ± 1.2	0.76 +/− 0.22	1491 ± 630.1	−10.9 ± 1.9	0.8 ± 0.18

Complete cell culture medium alone was measured to determine the effect (if any) of the serum proteins derived from either the horse serum (HS) or foetal bovine serum (FBS) components. Where RPMI 1640 medium supplements: 10% HS, 1% L-glutamine, DMEM 5546 medium supplements: 10% FBS, 1% L-glutamine, 1% P/S.

When comparing between the three technologies used to ascertain particle and agglomerate size there are differences. The SEM and DLS correlate at the higher concentration ranges. But at the low- to mid-concentration ranges the TEM analysis and DLS are more aligned. From the analysis it can be deduced that the agglomeration of this material is incredibly dynamic and appears to be subject to variation as the concentration increases. It does appear that the TEM and low concentrations of DLS show good concordance with the manufacturer stated size of >200 nm.

### Acellular dissolution

**Figure 2:**
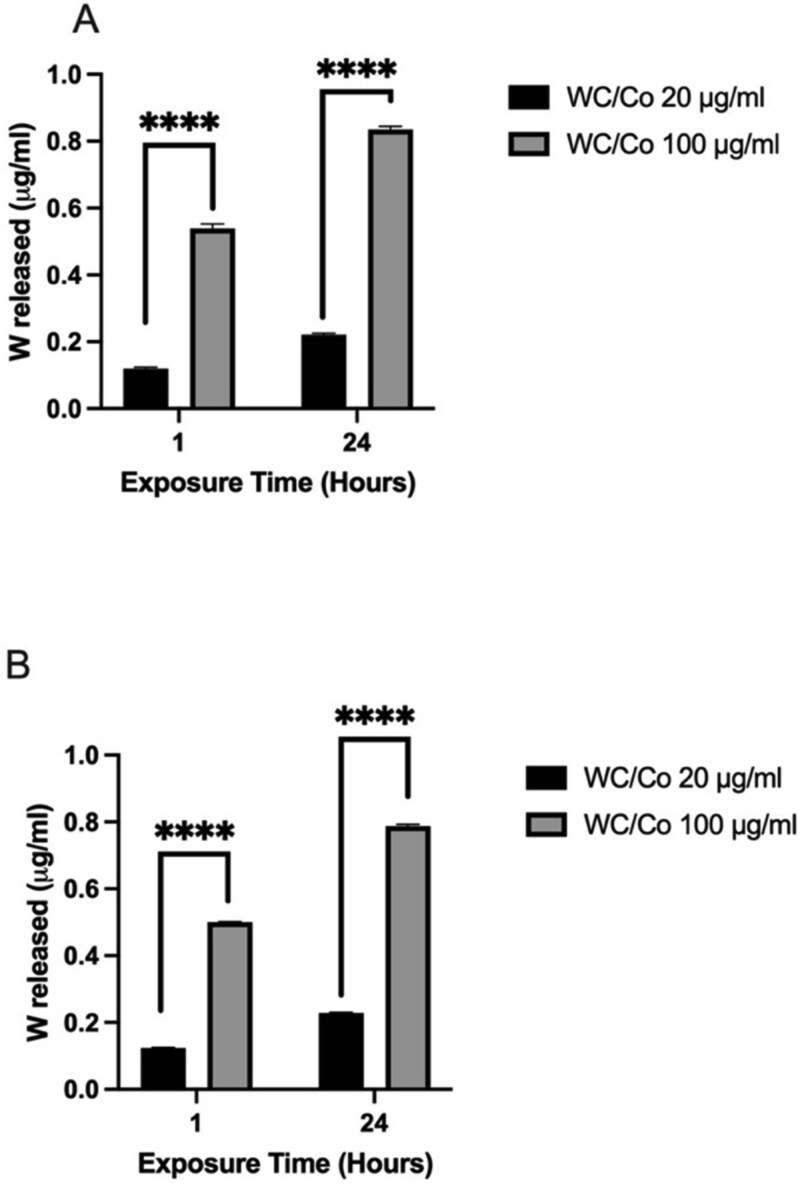
Release of tungsten ions from WC/Co nanoparticles dispersed in DMEM (A) and RPMI (B) complete cell culture medium. Two concentrations of 20 μg/ml (black bars) and 100 μg/ml (grey bars) were selected and tested over two time points of 1- and 24-hours. Data presented are the average of three replicates ± the SD. Significant differences between the control group and treatments are indicated by ^****^P ≤ 0.0001, (*n* = 3).

To ascertain the level of tungsten ion release from the nanoparticles into the suspension buffer (complete cell culture medium), ICP-OES was utilized. Whilst the concentration of tungsten ions is ~5x higher at 100 μg/ml as opposed to 20 μg/ml at both 1 and 24-hours, this is simply due to the concentration being 5x greater (Fig. 2). However, the maximum tungsten ion release (%) was observed for the concentration of 20 μg/ml after 24-hours which reached 1.11% and 1.14% in DMEM and RPMI, respectively. Thus at 20 μg/ml after 24-hours ~1.1% of the 20 μg/ml applied concentration of WC/Co nanoparticles was converted into tungsten ions. Interestingly, at 100 μg/ml after 24-hours the dissolution rate was lower at 0.84% and 0.79% in DMEM and RPMI, respectively. This lower dissolution rate which was observed may correlate to the fact that as concentration of the WC/Co nanoparticles increases, the agglomeration increases (evidenced by all three measurement techniques described earlier). One possible explanation for the greater dissolution of WC/Co nanoparticles into tungsten ions at lower concentrations is that there are fewer large agglomerates and potentially a greater monodispersed sampling of nanoparticles which are more readily able to break down into tungsten ions, a result which is further supported by the PI values in Table 1.

### *In vitro* cytokinesis-blocked micronucleus assay

**Figure 3:**
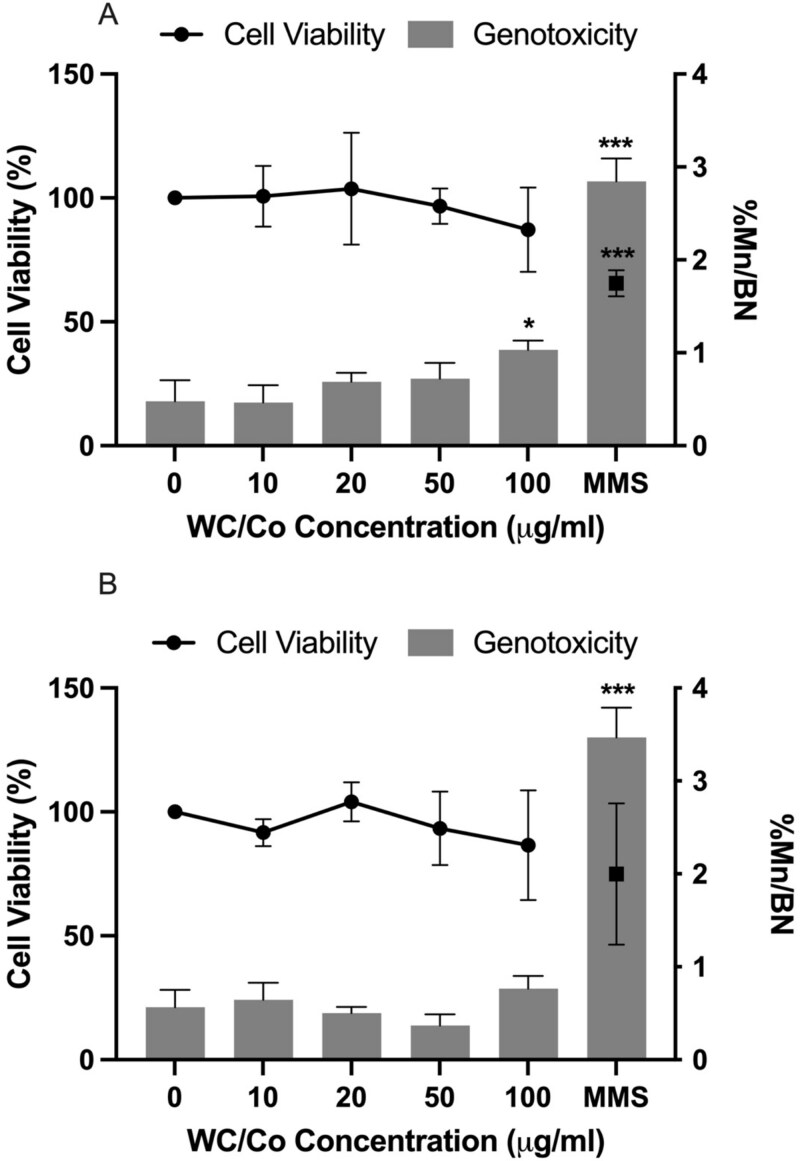
Cell viability and genotoxicity in TK6 (A) and V79–4 (B) cells following a 24-hour exposure to WC/Co and MMS as a chemical positive control. The data presented is the average ± the standard deviation (SD). Significant differences between the control group and treatments are indicated by ^*^P ≤ 0.05, ^***^P ≤ 0.001, (*n* = 3).

Following a 24-hour exposure to the test materials, the CBMN assay was performed to determine the cytotoxicity and genotoxicity in TK6 and V79–4 cells via RPD and the frequency of micronuclei in binucleated cells respectively (Fig. 3). The WC/Co nanoparticles did not induce any statistically significant cytotoxicity in either cell type; the chemical positive control, MMS, did reduce the TK6 cell viability to ~62%. Furthermore, the MMS control induced a statistically significant induction of micronuclei in binucleated cells with a 6-fold increase observed in TK6 and a 6-fold increase in V79–4 over background levels respectively. Crucially, the WC/Co nanoparticles generated statistically significant micronuclei frequencies in TK6 cells (only) at the highest test concentration of 100 μg/ml; this represented a 2-fold increase over background levels.

### *In vitro* mammalian cell gene mutation assay

**Figure 4:**
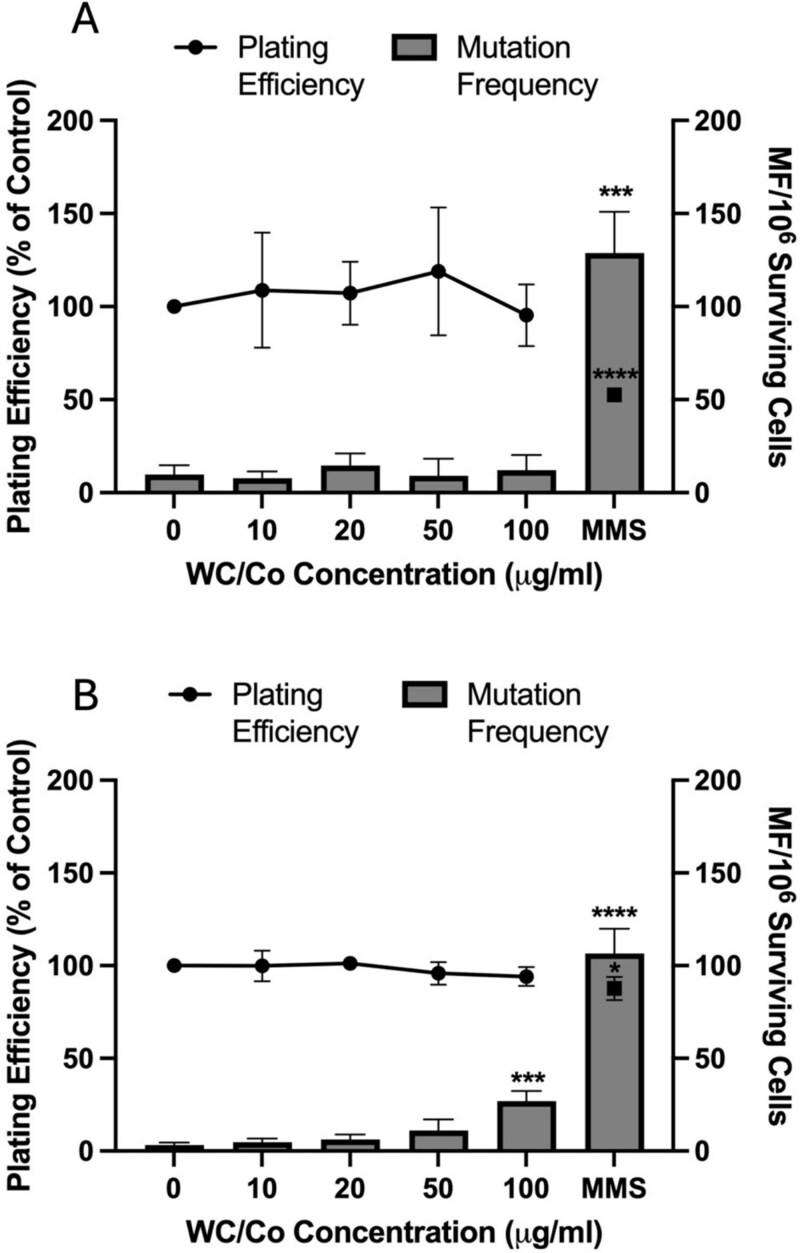
Cytotoxicity and mutagenicity of WC/Co nanoparticles following a 24-hour exposure to TK6 (A) and V79–4 cells (B). The data presented is the average ± the standard deviation (SD). Significant differences between the control group and treatments are indicated by ^*^P ≤ 0.05, ^***^P ≤ 0.001, ^****^P ≤ 0.0001, (*n* = 3).

Following a 24-hour exposure to the test materials, the *in vitro* mammalian cell gene mutation assay was performed to determine the plating efficiency and mutagenicity in TK6 and V79–4 cells (Fig. 4). The TK6 cells did not show any significant cytotoxicity or point mutations induction within the HPRT gene at any test concentration. The lack of cytotoxicity in the gene mutation test shows good concordance with the lack of cytotoxicity calculated in the CBMN assay (determined via different methods). The MMS chemical positive control however produced a highly significant response illustrated in Fig. 4A with a 14-fold increase over the background mutation frequency. The initial cytotoxicity data (denoted as PE0) and individual breakdown of the plating efficiency and mutation frequency from the two separate days in which the plates were scored can be viewed in the Supplementary Information (Fig. S1). To harmonize among the two different operating protocols used (one for suspension TK6 cells and one for adherent V79–4 cells) an average of the data was performed for the V79–4 cells and shown in Fig. 4B. Briefly, the initial cytotoxicity assessment represented by PE0 in the Supplementary Information, Fig. S1 (B) indicated there were no significant cytotoxic effects induced in V79–4 cells by the WC/Co nanoparticles, a small statistical effect was observed in cells exposed to MMS. A statistically significant increase in mutagenicity was observed at the highest test concentration of 100 μg/ml, in the absence of cytotoxicity. However, this response was substantially lower than the increase in mutation frequency induced by MMS in the V79–4 cells, which was accompanied by a minor but statistically significant drop in cell viability. Moreover, the MMS positive control proved reliably more potent as a mutagenic control than the damage induced by the WC/Co nanoparticles in V79–4 cells.

### Evaluation of strand breaks by the *in vitro* alkaline comet assay

**Figure 5:**
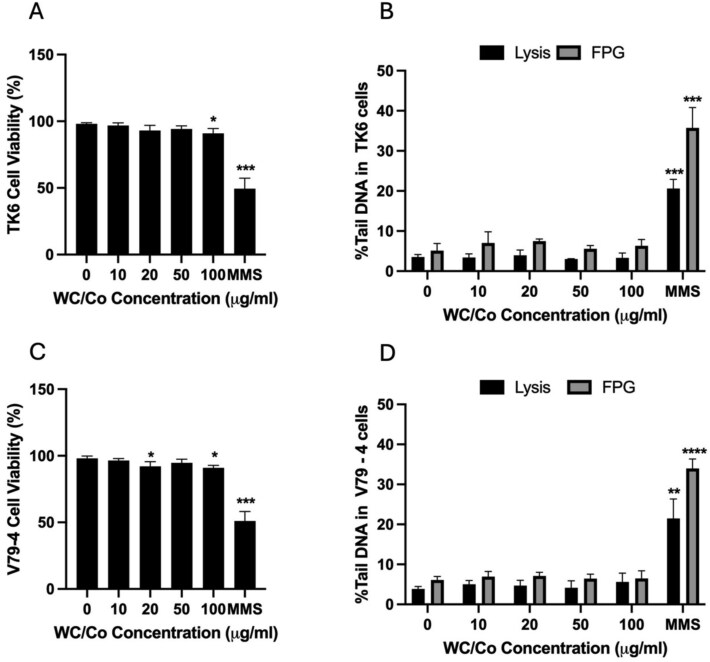
The cell viability and strand breaks in TK6 and V79–4 cells was measured by trypan blue exclusion and the comet assay respectively following a 24-hour exposure to MMS and WC/Co. The cell viability of TK6 and V79–4 cells are depicted in panels A and C, respectively. Panels B and D depict the %tail DNA in TK6 and V79–4 cells, respectively. Where strand breaks are shown as ‘FPG’ in the figures, this data refers to total strand breaks (SB + FPG) and does not represent net FPG-specific sites. The trypan blue exclusion data presented is the average ± the standard deviation (SD), whereas the DNA damage measured via the comet assay and FPG-modified comet assay is reported as the % of DNA in the comet tails (%tail DNA). The comet data presented is the average of the median ± the standard deviation (SD). Significant differences between the control group and treatments are indicated by ^*^P≤ 0.05, ^**^P ≤ 0.01, ^***^P ≤ 0.001, ^****^P ≤ 0.0001, (*n* = 3).

Following a 24-hour exposure to the test materials, the *in vitro* alkaline comet assay and modified, (FPG-treated) comet assay, was performed in parallel with Trypan blue exclusion (as a measure of cytotoxicity) to assess strand breaks and oxidative DNA damage in TK6 and V79–4 cells (Fig. 5). The cytotoxicity assessment run alongside the comet assay indicated statistically significant cytotoxicity was induced at 100 μg/ml in TK6 cells and at 20 and 100 μg/ml in V79–4 cells. This small reduction in viability (~8%–10%) is likely not evidence of a biologically significant response, however. The positive chemical control, MMS was able to consistently induce a statistically significant drop in cell viability to ~50% in both cell types. There were no statistically significant strand breaks (SBs) observed following nanoparticle exposure to either cell type, this includes observations made from the FPG-modified comet assay. The positive control of MMS generated highly significant SBs in both cell types, moreover whilst MMS is an alkylating agent—a noticeable increase in SBs were observed following enzymatic treatment with the FPG enzyme (Fig. 5B & D).

### Evaluation of nanoparticle-cell association and uptake

**Figure 6:**
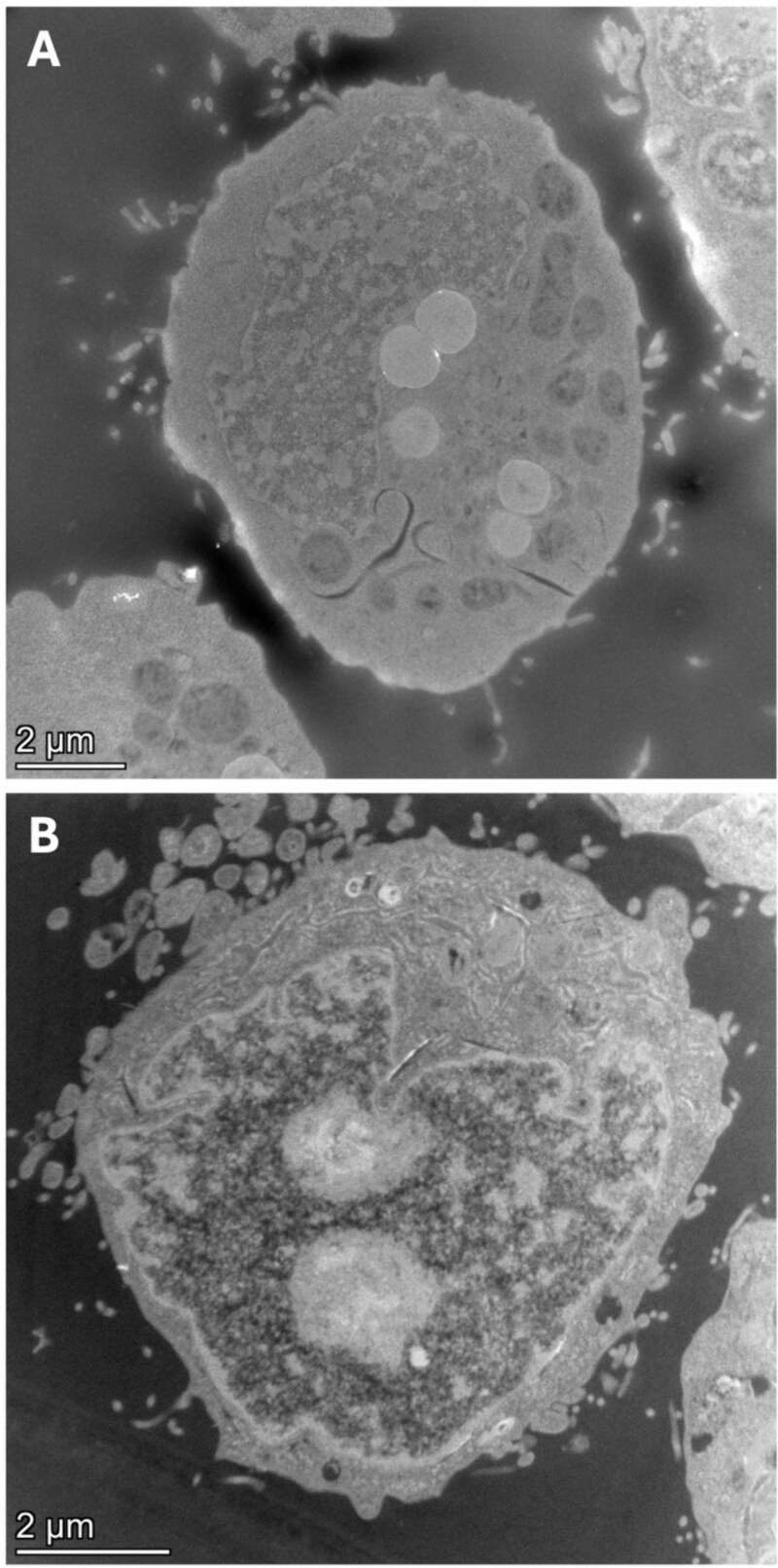
Transmission electron microscopy cellular uptake assessment in TK6 (A) and V79–4 cells (B). No cellular uptake was observed in TK6 or V79–4 cells following a 24-hour exposure to WC/Co nanoparticles. From each copper grid, 20 cells were imaged from three site areas of the resin section. A total of three grids were investigated (approximating to 180 cells). The images were captured using high-angle annular dark-field in scanning/transmission electron microscopy in S/TEM.

TEM was utilized following a 24-hour exposure of TK6 and V79–4 cells to WC/Co nanoparticles to qualitatively deduce if cellular uptake of the material was occurring. No cellular uptake of WC/Co nanoparticles was observed in TK6 cells or V79–4 cells at 20 μg/ml after surveying ~180 cells for each cell line (Fig. 6). It should be noted that there were limitations in the embedding and sectioning of cells at the highest test concentration of 100 μg/ml due to the hardness of the WC/Co material in comparison to the comparably soft resin sections. Sample sectioning on the ultramicrotome at 100 μg/ml introduced damage to the sections whereby the WC/Co was being removed from the resin. The WC/Co nanoparticles also proved difficult to remove from the suspension TK6 cells via centrifugation and PBS wash steps following exposure. Whilst no qualitative uptake was observed under these conditions it is important to consider this does not rule out uptake of WC/Co into these two cell lines. Given the small sampling of cells imaged compared to the total number of cells which underwent exposure and the subsequent genotoxicity which has been observed at 100 μg/ml it is likely that uptake could occur in these two cell lines.

## Discussion

Published data relating to the genotoxic response of WC/Co nanoparticles, specifically regarding the induction of micronuclei, suggests that under certain conditions dictated by cell type and concentration applied, this material could potentially be used as a positive particle control [1]. For instance, WC/Co nanoparticles induce a positive genotoxic response in isolated human leukocytes and rat lung epithelial cells [18, 19]. Moche *et al* have also shown positive data for the *in vitro* micronucleus test in L5178Y with WC/Co inducing statistically significant micronuclei in both short- and long-term exposures. The authors report that in long-term exposures of 24 hours followed by 20 hours of recovery the resulting data become overwhelmingly positive with a clear dose–response relationship [12]. It should be noted however that not all published data reflected a positive particle response; for example, a statistically significant response was not observed when HepG2 cells were exposed to WC/Co (8.25–33 μg/ml) [7, 20]. In the present study WC/Co induced a significant increase in micronuclei frequency in TK6 cells at the top concentration of 100 μg/ml. Thus, the implication is that WC/Co can only be applied as a positive particle control in certain cell types and typically at the highest test concentration, and it cannot be widely used for all cell lines.

As shown in the OECD GD No. 359, the predominant mode of action for WC/Co nanoparticles at 100 μg/ml appeared to be driven by centromere-negative micronuclei using fluorescent in-situ hybridization (clastogenicity) [7]. In their 2015 mechanistic study, Moche *et al* reported that after 4 hours of WC/Co exposure followed by a 20-hour recovery in L5178Y cells at 100 and 120 μg/ml there was statistically significant centromere negative responses. Also in this study, an increase in net FPG-sensitive sites in human lymphocytes were observed which suggests WC/Co particles induce genotoxicity through an oxidative stress-based mode of action [13]. In an independent investigation, Liu *et al* demonstrated conclusive evidence that WC/Co nanoparticles induced ROS (measured using the 2′-7′ dichlorodihydrofluorescein diacetate (DCFH-DA) assay) in BEAS-2B cells at concentrations as low as 5 μg/cm^2^, as early as 15 minutes into the exposure and increased up to the end exposure time of two hours [21]. ROS, and specifically the hydroxyl radical has been shown in the literature to damage both sugar and base components of DNA [22, 23]. The generation of ROS has specifically been linked to the dissolution of cobalt which (being a transition metal) can participate in Fenton-like chemical reactions in the presence of *in vitro* test systems [24, 25]. In a 2008 study by Fenoglio *et al*, the mechanism of action of WC/Co dusts contributing towards its classification of a group 2A material (probable human carcinogen) was investigated. The authors demonstrated WC/Co dusts induced oxidative damage to glutathione and cysteine thiol groups involving the generation of sulphur-centred radicals [26].

The capacity of WC/Co to induce mutagenic events has also been evaluated using the MLA-TK gene mutation test [12]. The results indicated a weak potential for WC/Co to induce point mutations *in vitro*. Even when significant rises in mutagenicity were observed this was related back to the high cytotoxicity limits induced, corresponding to a relative total growth of 10%–20%. In the present study, there was only a single statistically significant response which appeared in V79–4 cells at the highest test concentration of 100 μg/ml. This positive response however was not comparable in magnitude to the response observed following MMS exposure which was 10-fold greater than the untreated control group in the gene mutation test.

To provide an initial indicator of genotoxicity the *in vitro* alkaline comet assay was used in addition to the FPG-modified comet assay to detect purine-specific oxidative lesions. Whilst there are limited published examples of WC/Co being used in the comet assay, in 2017 Catalan *et al* did employ a combination of WC/Co (1 mg/mouse) and cyclophosphamide for an *in vivo* pharyngeal aspiration exposure and then followed up with comet and micronucleus experiments. The findings showed statistically significant %Tail DNA (in lung and BAL cells) and 3.4-fold increase in micronuclei in polychromatic erythrocytes [27]. In the present study however, there was no significant increases in %Tail DNA in exposed cells. In a 2015 study, WC/Co nanoparticles were exposed to human lymphocytes (40–120 μg/ml) and to L5178Y mouse lymphoma cells (30–100 μg/ml). The results of the FPG-modified comet assay conducted by Moche et al showed no increase in net FPG-sensitive sites in comparison to the negative control in human lymphocytes. In the L5178Y cells a slight increase was observed at 80 and 100 μg/ml, however this was not statistically significant [13]. In the present study there was a marked increase in net-sensitive FPG sites following MMS exposure indicative of additional damage relating to oxidative lesions. One theory is this may be because of other imidazole-specific damage being incurred which FPG is then able to act upon, such as 8-oxoguanine lesions. There is evidence in the literature that alludes to this capacity of MMS to induce oxidative damage, and the subsequent elevated SBs observed following FPG incubation [28–32]. In the present study, the Trypan blue exclusion assay was used as a measure of cell viability, primarily to avoid nanoparticle interference (which is sometimes observed with absorbance- and fluorescence-based techniques), however it should be noted that there are other viability measures which can be utilized in conjunction with the comet assay. The colony forming efficiency assay for example would be an alternative approach to avoiding nanomaterial interference as it provides an interference- and label-free methodology for measuring cell viability and colony forming efficiency [33, 34].

To determine if the release of metal ions may have been involved in driving the genotoxic responses observed, dissolution of WC/Co nanoparticles was evaluated by ICPOES. As demonstrated in Fig. 2, only a small fraction of the applied concentration of WC/Co nanoparticles breakdown to release tungsten ions; ~1.2%, at 20 μg/ml. Therefore, the release of tungsten ions is unlikely to be driving the genotoxic responses which are only observed at the highest test concentration of 100 μg/ml for both the CBMN assay and gene mutation test. Concerning the presence of cobalt (and its possible contribution to a genotoxic response), given that the WC/Co formulation is only 8% by weight in cobalt it may not be the driving factor in the observed genotoxic response, particularly given the insoluble nature of the formulation, which means the cobalt is not likely to take on a readily soluble form. The genotoxicity of cobalt metal and particles has been associated with conflicting outcomes in the scientific literature. For example, Kirkland *et al.* [35] showed that *in vitro* studies on cobalt compounds induced chromosomal damage but not mutations and that the damage may be a consequence of oxidative stress. In contrast, the data for *in vivo* chromosomal damage were negative [35]. Caution should be taken however when comparing the effects of cobalt metal powders and fine cobalt metal powders to true nanoparticulate cobalt. Cobalt ion release by nanoparticulate cobalt has not been extensively investigated as compared to the ion release by soluble organometallic compounds (salts) such as cobalt octoate or cobalt sulfate heptahydrate [35].

Whilst no cellular uptake was observed at 20 μg/ml it is important to note that only a small number of cells can be analysed using TEM given the extremely low throughput of the technique. Additionally, the hardness of the WC/Co particles in comparison to the resin sections introduces limitations to the concentrations we can successfully process. Therefore, whilst uptake into TK6 and V79–4 cells was not observed in this study, the possibility of WC/Co uptake into the cells cannot be ruled out, particularly where a genotoxic response has been observed. There are alternative techniques which can offer some insight into cellular association, one such technique being laser-ablation inductively coupled plasma mass spectrometry (LA-ICP-MS). This approach has previously been used to demonstrate that V79–-4 cells show a concentration-dependent signal increase when exposed to WC/Co at 30, 60, and 100 μg/ml [1]. Whilst this technique cannot qualitatively prove uptake due to the destruction of the tissue, it can offer a quantitative measure of cellular association. Thus, one potential avenue for future studies looking to evaluate uptake is to combine such techniques (TEM with LA-ICP-MS) in an effort to determine cellular capacity for internalizing nanomaterials.

In conclusion, this study has demonstrated that WC/Co nanoparticles may be used as a positive particulate control for genotoxicity testing in TK6 cells when using the CBMN assay, and for V79–4 cells in the gene mutation assay, but only at the top concentration of 100 μg/ml when suspended using the NANoREG dispersion protocol. Future studies which employ WC/Co nanoparticles in their study design should be aware of the specific conditions required to generate a positive response, including; the assay used, the cell line selection and the concentration applied. The latter of which should not exceed 100 μg/ml to avoid assay interference and departure from physiological relevance as defined in the OECD GD No.359 [7].

## Supplementary Material

Supplementary_Information_geaf021

## Data Availability

The data generated in this manuscript are available from the corresponding author upon reasonable request.
