# Supplementary material for: Tungsten carbide–cobalt can function as a particle positive control for genotoxicity in vitro in specific cell lines
Source: Mutagenesis. 2025 Oct 3;40(5-6):592–602. doi: 10.1093/mutage/geaf021 (PMC12720410; doi:10.1093/mutage/geaf021)
Supplement: Supplementary_Information_geaf021 [file supplementary_information_geaf021.docx]

**Supplementary Information**

Tungsten carbide-cobalt can function as a particle positive control for genotoxicity *in vitro* in specific cell lines

Michael J Burgum^1^, Stephen J Evans^1^, Ilaria Zanoni^2^, Magda Blosi^2^, Gareth Jenkins^1^, Shareen H Doak^*1^

^1^ *In Vitro* Toxicology Group, Faculty of Medicine, Health and Life Sciences, Institute of Life Sciences, Swansea University Medical School, Singleton Park, Swansea, SA2 8PP, Wales, UK.

^2^ CNR-ISSMC, National Research Council of Italy-Institute of Science, Technology and Sustainability for Ceramics, Faenza, Italy.

*Corresponding Author

E-mail address: s.h.doak@swansea.ac.uk


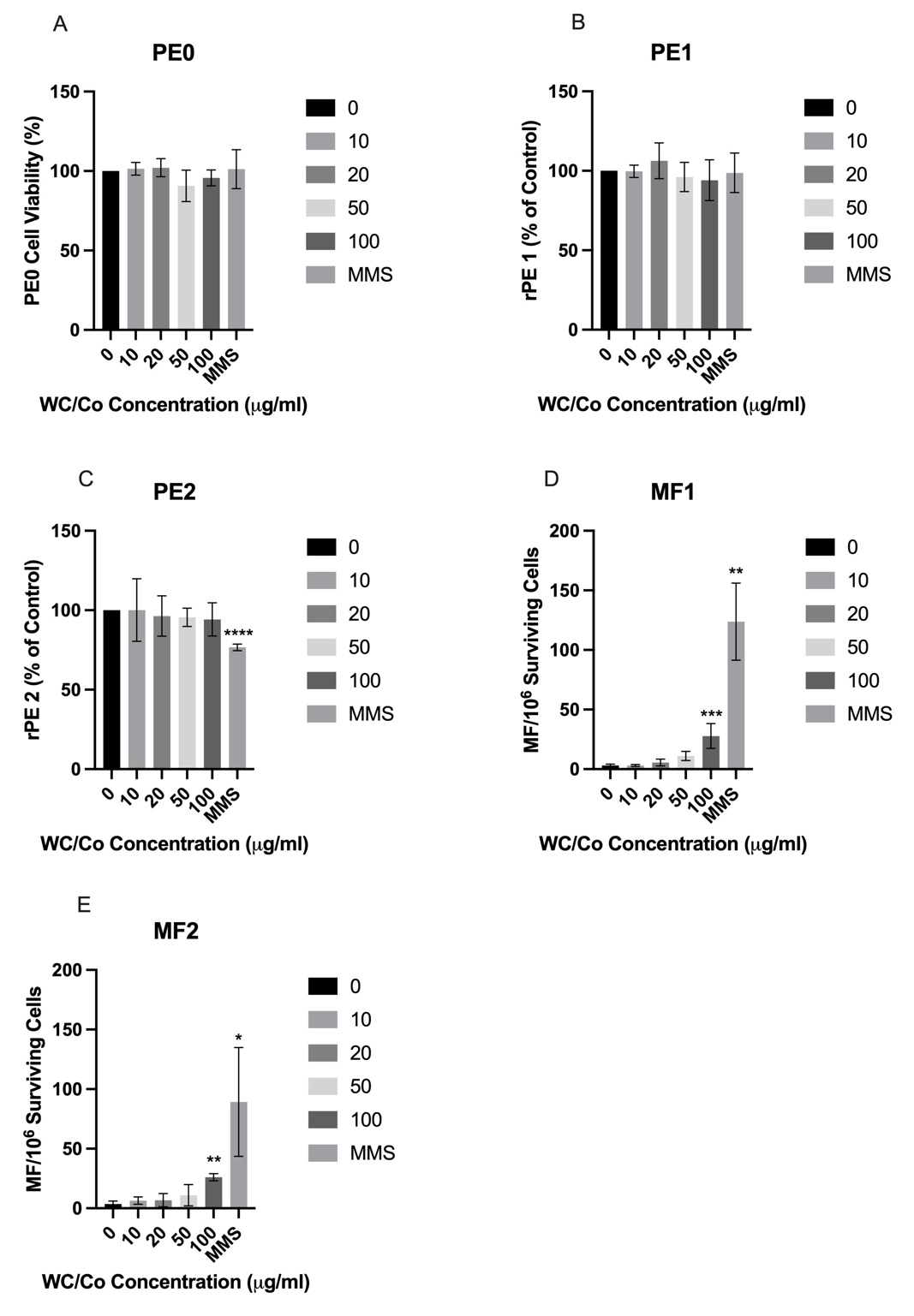


**Figure S1**. Cytotoxicity and mutagenicity of WC/Co following a 24-hour exposure whereby initial cytotoxicity is represented by PE0 (A). The first mutation frequency and concordant cytotoxicity are represented by rPE1 and MF1 (B). The second plating for mutation frequency and concordant cytotoxicity are represented by rPE2 and MF2 (C). The data presented is the average +/- the standard deviation (SD). Significant differences between the control group and treatments are indicated by ^*^*p*≤0.05 ^**^*p*≤0.01 ^***^*p*≤0.001 ^****^*p*≤0.0001, (*n*=3).


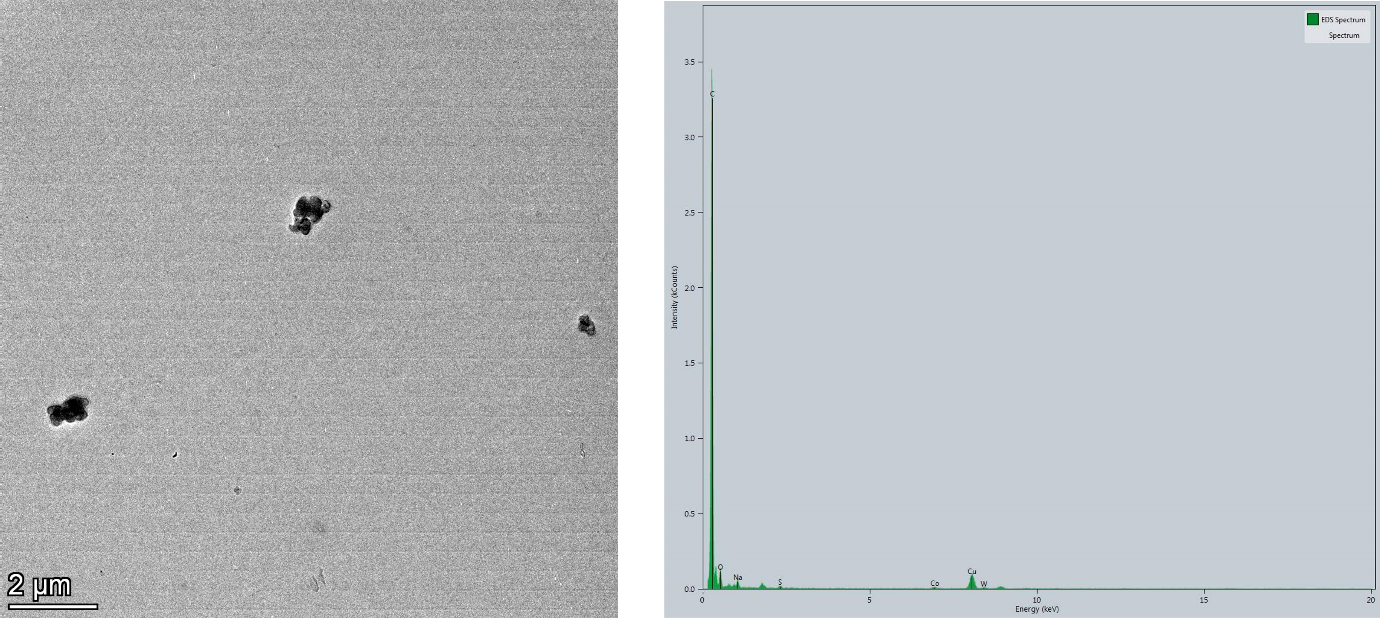


**Figure S2**. Representative TEM electron micrograph and EDS particle analysis.
